# Interferon-Inducible Protein 16 (IFI16) Has a Broad-Spectrum Binding Ability Against ssDNA Targets: An Evolutionary Hypothesis for Antiretroviral Checkpoint

**DOI:** 10.3389/fmicb.2019.01426

**Published:** 2019-07-04

**Authors:** Tara Patricia Hurst, Amr Aswad, Timokratis Karamitros, Aris Katzourakis, Adrian L. Smith, Gkikas Magiorkinis

**Affiliations:** ^1^Department of Zoology, University of Oxford, Oxford, United Kingdom; ^2^Department of Life Sciences, School of Health Sciences, Birmingham City University, Birmingham, United Kingdom; ^3^Laboratory of Medical Microbiology, Department of Microbiology, Hellenic Pasteur Institute, Athens, Greece; ^4^Department of Hygiene, Epidemiology and Medical Statistics, Medical School, National and Kapodistrian University of Athens, Athens, Greece

**Keywords:** endogenous retrovirus, IFI16, evolution, checkpoint, ssDNA

## Abstract

Human endogenous retroviruses (HERVs) are under genomic and epigenetic control but can be expressed in normal tissues, producing RNA transcripts some of which are translated. While it has not been demonstrated experimentally in modern humans, cDNA copies from HERV RNA (namely HERV-K HML-2 or HK2) were produced after the human-chimp split and until at least 250,000 years ago. We were interested in determining if such cDNA could be a ligand for pattern recognition receptors (PRRs) of the innate immune response. The AIM-2-like receptors for DNA, interferon-γ-inducible protein 16 (IFI16) and Cyclic GMP-AMP synthase (cGAS) were candidate PRRs. IFI16 can detect cDNA produced during HIV-1 replication, causing increased T cell death. While HIV-1 has emerged relatively recently as a human pathogen, the cDNA functionality of IFI16 could have been selected for during the course of human evolution. Here we present a novel hypothesis that the products of reverse transcription of HK2, which has been proliferating in the genome of human ancestors for 30 million years, could interact with IFI16. In support of our hypothesis, we provide preliminary data showing that IFI16 (but not cGAS) interacts with synthetic single-stranded HK2 oligos corresponding to the first product of reverse transcription. Further, we show that ssDNA detection by IFI16 has variability with respect to sequence features but is not dependent on strong secondary structures mimicking dsDNA. Among the HK2 oligos, IFI16 interacts more intensely with those derived from LTRs, suggesting these oligos have undetermined structural features that allow IFI16 to bind with greater affinity. Further, cells with stem cell features that naturally allow HK2 expression were found to express many components of the innate immune system including cGAS but not IFI16. Based on the presented preliminary data we further postulate another hypothesis: that the IFI16 functionality in human cells has been acting as “second-line” defense to control abnormal HK2 replication in somatic tissues. The absence of this protein in stem cells and a stem cell line could permit these cells to express HERVs which contribute to stem cell identity. Finally, we also comment on potential studies that could support or refute our hypothesis.

## Introduction

Retroviral proliferation in somatic tissues can be detrimental to the host. Both endogenous and exogenous retroviruses have been shown to be responsible for tumorigenesis and immunodeficiencies (Coffin et al., 1997). We have shown that recent (i.e., within the last 10 million years) endogenous retrovirus (ERV) proliferation activity is negatively correlated with mammalian body size, suggesting a negative impact of retroviral activity within somatic tissues (Katzourakis et al., 2014). Crucially, ERVs may survive in the long term by constant germline proliferation (Magiorkinis et al., 2015), suggesting that survival of the host until reproductive age is advantageous for ERVs. It is thus reasonable to hypothesize that the relatively higher activity during early life events (allowing for proliferation in the germline) compared to a lower activity in somatic tissues (minimizing the pathogenic effects) is advantageous for both the host and the ERV. Further to this, human endogenous retroviruses (HERVs) are expressed in germ cell lines, with viral particles budding from cell membranes (Bieda et al., 2001). RNA-Seq analysis of a teratocarcinoma cell line (Tera-1) revealed the extent of HK2 expression from multiple loci and the activity of many of the LTRs in driving expression (Bhardwaj et al., 2015).

The expression of HERVs in somatic tissues is regulated by numerous mechanisms, including epigenetic silencing through histone acetylation, methylation and chromatin packing (Hurst and Magiorkinis, 2017). HERVs are prevalent in the genome but are mostly defective proviruses or solo long terminal repeats (LTRs) (Belshaw et al., 2007) as a result of mutagenesis or recombinatorial deletion during co-evolution. Despite these control mechanisms, HERV expression in somatic cells has been demonstrated. A microarray study showed that almost one-third of HERV loci are transcribed (Pérot et al., 2012). Expression of HERVs or derepression of HERV LTRs has been hypothesized to be pathogenic. For example, the derepression of HERV LTRs could lead to activation of otherwise silent oncogenes, a process referred to as “onco-exaptation” (Babaian and Mager, 2016). Thus, a “second line” of defense could be beneficial for the human host, one that would detect the presence of abnormal HERV nucleic acids in somatic tissues.

It has been hypothesized that the products of HERV replication could be detected by pattern recognition receptors (PRRs) of the innate immune response. PRRs detect molecular signatures of pathogens, such as distinct nucleic acid structures or modifications. For example, the presence of HERV nucleic acids could be a trigger for specific PRRs, much like the products of an exogenous viral infection. In this manner, the HERVs could be acting as danger signals (Hurst and Magiorkinis, 2015), activating the innate immune system to endogenous ligands that should not normally be present or allowed to accumulate within cells. For murine retroelements, for example, it was found that cDNA can accumulate in cells with defective three prime exonuclease 1 (TREX1) (Stetson et al., 2008). Defective TREX1 is associated with aberrant immunity in at least one condition, Aicardi-Goutières Syndrome (AGS) (Lehtinen et al., 2008). Thus, if retroelement cDNA would be produced it could lead to physiological outcomes including interferon (IFN) and pro-inflammatory cytokine expression. While it has been hypothesized, the ability of HERV nucleic acids to act as a ligand for a PRR has not been shown.

Here we postulate the hypothesis that an innate immune response to HERV cDNA is likely to take place by analogy to studies showing detection of HIV-1 reverse transcripts. If produced, HERV cDNA would be in the cytosol wherein numerous PRRs specific to DNA reside, including a member of the AIM2-like receptor (ALR) family, IFNγ-inducible protein 16 (IFI16), as well as cyclic GMP-AMP synthase (cGAS). Interestingly, both IFI16 and cGAS have been found to detect HIV-1 cDNA though there seem to be distinct ligands detected by each receptor. For cGAS, it could detect Y-form cDNA containing unpaired guanosines flanked by short dsDNA sequences (Herzner et al., 2015). In contrast, IFI16 could bind to both ssDNA and dsDNA, the sequential products of reverse transcription of HIV-1 (Monroe et al., 2014). In that study, IFI16 was found to be critical for the detection of cDNA from abortive HIV-1 infections in CD4+ T cells, leading to T cell death (Monroe et al., 2014). Since one function of IFI16 is the induction of pyroptosis, a role in T-cell death following HIV-1 cDNA detection is not surprising. In addition, a study using cDNA oligos derived from the HIV-1 LTR found that one of these (ssDNA1) could induce a robust IFN response (Jakobsen et al., 2013). Thus, multiple physiological outcomes of IFI16 ligation are possible.

Detection of HERV cDNA by these PRRs would require its physical association with their DNA-binding domains. Both IFI16 and cGAS bind to the sugar-phosphate backbone and thus detection of DNA by these PRRs is thought to be sequence-independent (Dempsey and Bowie, 2015). Cytosolic dsDNA is the ligand for cGAS although it can also sense reverse transcribed HIV-1 DNA (Chan and Gack, 2016). Indeed, it has also been shown to bind to HIV-1 cDNA in a sequence-specific manner and to detect stem-loop structures (Herzner et al., 2015). The synthesis of the second messenger 2′,3′-cGAMP by cGAS (Zhang et al., 2013) is critical for the activation of the adaptor, stimulator of IFN genes (STING), and the subsequent production of IFN in response to aberrant DNA (McFadden et al., 2017). The other cytosolic receptors, including IFI16, are thought to have redundant roles or to somehow feed into the cGAS-STING axis (Jakobsen and Paludan, 2014), possibly by amplifying the signal of cGAS (Jønsson et al., 2017). The hematopoietic expression interferon-inducible nature and nuclear localization (HIN) domains of IFI16 are responsible for binding to dsDNA in a sequence-independent manner, triggering receptor activation (Jin et al., 2012). In addition, IFI16 has been shown to detect ssDNA molecules, particularly those with secondary structures such as those that resemble dsDNA (Sparrer and Gack, 2015), as well as quadruplex DNA (Hároníková et al., 2016), indicating that multiple ligands could exist for this receptor.

We further postulate the hypothesis that cytosolic DNA PRRs could be a second line of defense against HERV activity. To provide some preliminary data in support of this hypothesis, we explore the roles of two of the most well-studied cytosolic PRRs, IFI16 and cGAS, in sensing HERV ssDNA. Since HERV-K HML-2 (HK2) has been the only HERV that proliferated in the human genome, we focus on the potential interaction of HK2 ssDNA with cGAS and IFI16. We employed a candidate approach to assess the ability of the cytosolic DNA receptors to bind to synthetic HERV oligos. We show that IFI16 interacts with HK2 ssDNA, even with scrambled ssDNA, suggesting a well-established role for the non-specific detection of retrovirally produced DNA in the cytosol. We then show that IFI16, but not cGAS, interacts with HK2 ssDNA derived from multiple genomic locations. We also show that in a teratocarcinoma cell line (NCCIT), which has an embryonic stem cell (ESC) phenotype, HK2 expression is high. In NCCIT cells, there is a remarkable absence of IFI16 although cGAS and other innate immune components are present. The activity of HERVs in this cell line suggests the absence of one or more factors that would normally control HERV expression; one such restraint could be IFI16. Based on our preliminary date, we postulate the hypothesis that IFI16 has been an evolutionarily preserved defense against retroviral activity in somatic tissues, while at the same time allowing an efficient proliferation in the germline. More studies are warranted to confirm or refute the hypotheses presented here, and more importantly explore the depth, extent and mechanistic properties of the role of IFI16 in controlling retroviral proliferation.

## Materials and Methods

### Cell Culture

The Jurkat E6.1 T cell line was purchased from the European Collection of Authenticated Cell Cultures (ECCAC; Porton Down, United Kingdom) and the teratocarcinoma cell line NCCIT was obtained from the American Type Culture Collection (ATCC, LGC Standards, Teddington, United Kingdom). Both cell lines were maintained in RPMI-1640 medium supplemented with 10% FBS (Sigma-Aldrich), 1% penicillin-streptomycin and 2 mM L-glutamine (Life Technologies). The cells were incubated in a humidified atmosphere at 37°C with 5% CO_2_ and cultured as described below. Jurkat cells were passaged twice per week at a ratio of 1:5. The NCCIT cells were allowed to grow to 100% confluence in a T75 flask, with sub-culturing every 4 days. Two days after seeding a new flask, the spent media was replaced with fresh media due to the elevated respiration rate in this cell line.

### Oligo Design

**FIGURE 1:**
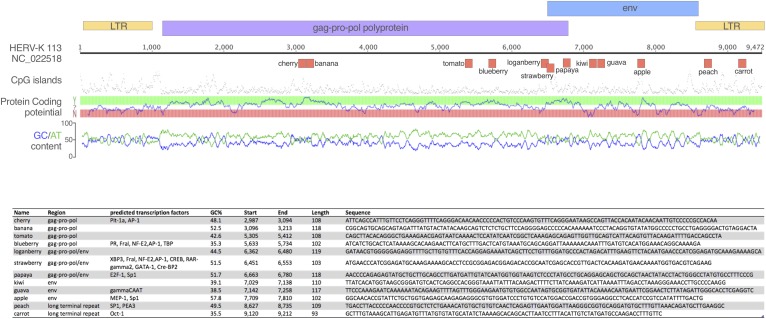
Oligonucleotides tested in the study. The schematic depicts the oligos tested in the study mapped onto the HERV-K113 genome. Above the ruler, the colored boxes represent the retroviral ORFs and LTR regions; below are the oligos, which are named for ease of reference. Corresponding statistics for each oligo are presented in the table, including the sequence of each oligo.

We designed oligos to use in biotin-streptavidin pulldowns to investigate their interaction with PRRs. The ssDNA1, ssDNA2 and ssDNA3 oligos are published sequences derived from the HIV-1 LTR (Jakobsen et al., 2013). Based on one study (Brázda et al., 2012), we hypothesized that the structure of the oligos was critical and so analyzed the structure of ssDNA1, the functional oligo described by Jakobsen et al. (2013), using mFOLD (Zuker, 2003). Next, we used a sliding window analysis of a consensus sequence for HK2 *env*, assessing 100 mer oligos using mFOLD for sequences with stem-loop structures similar to that of ssDNA1. From this, we selected the oligo labeled HK2 *env*. Subsequently, we scrambled this sequence (HK2 *env* scrambled) and mutated bases we hypothesized to be critical to the structure (HK2 *env* mut). Next, we performed the same analysis of folding of 100 mer sequences from the HK113 LTR (GenBank: AF387847.1) and *env* (Genbank NC_022518) and derived the oligos so-named. We then designed a panel of oligos spanning the genome of HERV K113 (NC_022518). Regions were selected from the *gag*, *pol* and *env* ORFs, as well as the LTR. Sequences ∼100 bp long were chosen to vary in GC content, CpG islands, predicted transcription factor motifs and relative distance from one another. The GC-content sliding window size was 48 bp. Transcription factor prediction was performed using the EMBOSS tool tfscan and the oligo sequences were visualized using Genious 8.1.3 and Pixelmator 3.6 (Figure 1). These oligos were given names rather than base pair ranges for ease of reference. Finally, a random highly GC-rich 100 mer oligo (GC59) was also synthesized. Oligo sequences are in Figure 1 and Table 1. The oligos were synthesized with a biotin tag added to the 3′ end and a spacer triethylene glycol (TEG) unit to prevent steric hindrance (Sigma-Aldrich).

**Table 1:** Sequences of HERV oligos.

Oligo Name	Sequence
**HK2 *env* oligo 350**	TCATTAAAATTTAGACCTAAAGGGAAACCTTGCCCCAAGGAAATTCCCAAAGAATCAAAAAATACAGAAGTTTTAGTTTG GGAAGAATGTGTGGCCAATA
**HK2 *env* 350 (mutated bases 45**–**51)**	TCATTAAAATTTAGACCTAAAGGGAAACCTTGCCCCAAGGAAATagggtttGAATCAAAAAATACAGAAGTTTTAGTTTGGGA AGAATGTGTGGCCAATA
**HK2 *env* 350 scrambled_2**	GATATGAACCAAATTAACCGAATGTAGGTAAGAAAAGGTCTACTAAATACCTATGACATTCAAGTGGGCTAGAAGCAATAA ATTTAAAATTCGCGACGTC
**HERV-K113 5**′ **LTR bases 1**–**100**	AAAGTAATTAGGGGTCTATCATGAGTACCAGACAAGTATAAACAAAATTTGCATGGGGAGATTCAGAACCAAAAGCCAT CGATTGTACAGTAATAAATTC
**HERV-K113 *env* 1**–**100**	ATGAACCCATCGGAGATGCAAAGAAAAGCACCTCCGCGGAGACGGAGACACCGCAATCGAGCACCGTTGACTCACA AGATGAACAAAATGGTGACGTCAG
**GC59**	GCTGTTGCGGCCCGAGAGGCTGAGTGATCTTCGGTTTTGAATCTGCCGTCTGCAGGGCGTACTGCGGTAGCGGACG GAGCTGGTGGTATCACGTAACACT

*These oligos are either based on the HK2 env consensus sequence that we also used to design primers and a probe for qPCR (HK2 env oligo 350, HK2 env oligo 350 mut, HK2 env oligo 350 scrambled) or on the HK113 consensus sequences from GenBank (HK113 LTR, HK113 env). A random scrambling of HERV sequences produced a GC-rich oligo (GC59) which was found to interact strongly with IFI16 and so was used as a positive control for the oligo–IFI16 interaction.*

### Biotin-Streptavidin Pulldown and Immunoblotting

We empirically determined that 2 × 10^6^ Jurkat cells would give sufficient material for pulldowns with three separate oligos. The required number of cells was harvested during routine culture of Jurkat cells and pelleted by centrifugation at 1000*g*. The medium was removed and the cells were either used immediately or the pellets stored at –20°C until use.

The Jurkat cell pellets were lysed in a gentle lysis buffer (137 mM NaCl, 20 mM Tris–HCl (pH 7.4), 1 mM EDTA, 0.5% v/v Triton-X 100), supplemented with HALT protease inhibitor cocktail and EDTA (Thermo Fisher Scientific) to a 1× final concentration and 60 mM n-Octylglucoside (Cayman Chemicals) immediately prior to use. Cells were lysed in 500 μL lysis buffer for 10 min on ice with physical disruption by pipetting, followed by centrifugation at 1000*g* for 10 min at 4°C. The supernatant was reserved for the pulldown. Of this, 50 μL was saved for a direct lysate sample; it was mixed with SDS-PAGE sample buffer and stored at –20°C for later analysis. The remaining 450 μL was pre-cleared for 1 h at 4°C with 25 μL of pre-washed streptavidin beads, which were then pelleted by centrifugation.

The pre-cleared lysate was then divided into four equal volumes and 2 μM of a single biotinylated oligo was added to each. The samples were incubated for 3 h at 4°C with constant mixing. Following this, 20 μL of fresh pre-washed beads were added to the lysates and they were incubated at 4°C for a further 1 h with constant mixing. The beads were then pelleted by centrifugation (10000*g* for 2 min at 4°C), washed three times with 0.5 mL of lysis buffer and then 30 μL of 2× SDS sample buffer was added. The samples were boiled for 5 min at 95°C, chilled on ice, centrifuged briefly and stored at –20°C for later gel analysis.

The pulldowns and whole cell lysates were analyzed with SDS-PAGE following a published method (Sambrook et al., 1989), using pre-cast 4–20% Tris-tricine gels (Bio-rad) and including ColourBurst protein mass marker (Sigma-Aldrich). The gel was run at 80 V for 1.5–2 h, then transferred to Immobilon-FL PVDF membrane (Merck Millipore) using the wet transfer system (Bio-Rad) with buffer containing 20% methanol. Following blocking in Starting Block (Thermo Fisher Scientific) for 1 h, the primary antibody was added and allowed to incubate overnight with continuous rocking at room temperature. The mouse monoclonal antibody to IFI16 (clone 1G7) was from Santa Cruz Biotechnology Inc. (RRID:AB_627775). A rabbit polyclonal antibody to cGAS was purchased from Merck Millipore. Secondary HRP-labeled anti-mouse and anti-rabbit antibodies were from Perkin-Elmer. Finally, the blot was washed and secondary antibody was added for 1 h at room temperature.The blots were developed using Novex enhanced chemiluminescence (ECL) from Life Technologies and a G:Box dark room (Syngene).

### Bioinformatics/RNAseq Analysis

Raw RNAseq reads (SRA: SRX037079, runs: SRR089638/ SRR094918) were mapped against the Human Transcriptome reference (v.GRCh38.rel79) using the TOPHAT pipeline (Trapnell et al., 2012) to calculate genes abundances. A threshold of 10 tpm was set for basal transcription activity (<10 tpm). Genes with more than 10 tpm in both datasets were considered active (Kröger et al., 2013). The rationale for the 10 tpm cut-off for increased gene expression is consistent with the approach of others in analyzing RNA-Seq data (Carninci, 2010; Kim et al., 2013; Kröger et al., 2013).

## Results

### IFI16 Can Bind to HIV-1 ssDNA Oligos With Variable Intensity

**FIGURE 2:**
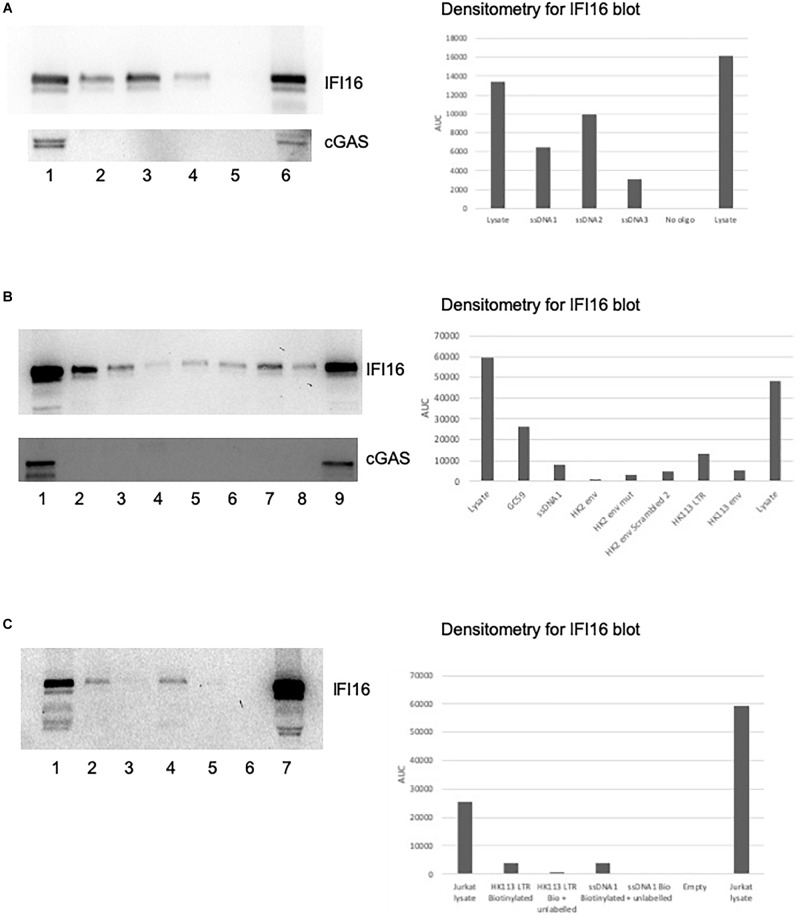
IFI16 binds to oligos from HIV-1 and HERV-K. Pre-cleared Jurkat cell lysates were incubated with biotin-labeled oligos, as described in the section “Materials and Methods.” The pulldowns were analyzed by SDS-PAGE and western blotting first for IFI16, followed by reprobing the same blot for cGAS. **(A)** The ssDNA 1, 2 and 3 oligos (Jakobsen et al., 2013) were examined for their interaction with IFI16 (top panel) and cGAS (bottom panel). The lanes were loaded with input lysate or pulldowns with oligos as indicated: (1) input lysate, (2) ssDNA1, (3) ssDNA2, (4) ssDNA3, (5) no oligo control and (6) repeat of the input lysate. **(B)** A HK2 oligo from the consensus sequence, as well as the HK113 sequence, were analyzed for interaction with IFI16 (top panel) and cGAS (bottom panel). The lanes were loaded with input lysate or pulldowns with oligos as indicated: (1) input lysate, (2) GC59, (3) ssDNA1, (4) HK2 *env*, (5) HK2 *env* mut, (6) HK2 *env* scrambled 2, (7) HK113 LTR, (8) HK113 *env* and (9) a positive input lysate from a previous experiment as a blotting control. **(C)** In this competition assay, the biotinylated ssDNA1 and HK113 LTR oligos were used in pulldowns with and without an untagged version of the same oligo. The lanes were loaded as follows: (1) input lysate, (2) HK113 LTR biotinylated oligo only, (3) HK113 LTR biotinylated and unlabeled oligos, (4) ssDNA1 biotinylated oligo only, (5) ssDNA1 biotinylated and unlabeled oligos, (6) empty lane and (7) repeat of the input lysate. Densitometry was performed on the IFI16 blots by measuring the area under the curve (AUC) with Image J.

**FIGURE 3:**
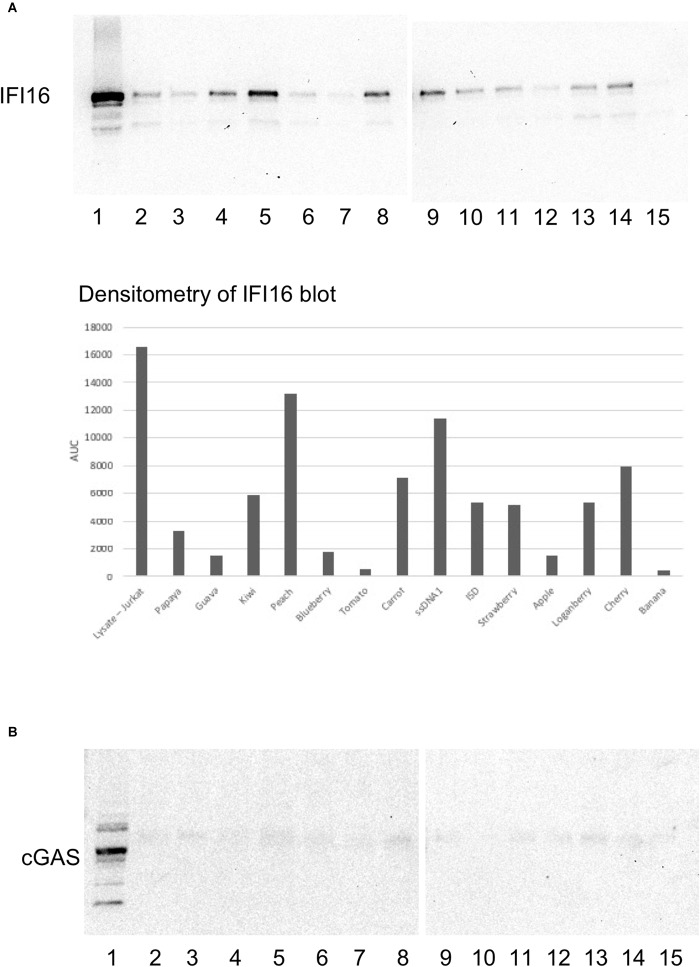
IFI16 binds to HERV-K oligos with variable affinity. Oligo sequences spanning the HK2 genome were synthesized and biotinylated. Pre-cleared Jurkat cell lysates were incubated with the biotinylated oligos, as described in the section “Materials and Methods.” The pulldowns were analyzed by SDS-PAGE and western blotting first for IFI16 **(A)**, followed by reprobing the same blot for cGAS **(B)**. The lysates or oligos in each lane are: (1) lysate (2) Papaya (3) Guava (4) Kiwi (5) Peach (6) Blueberry (7) Tomato (8) Carrot (9) HIV-1 ssDNA1 (10) ISD (11) Strawberry (12) Apple (13) Loganberry (14) Cherry (15) Banana. For oligo sequences, see Figure 1. ISD, the immunostimulatory domain from *Listeria* (Hansen et al., 2014). Densitometry was performed on the IFI16 blots by measuring the area under the curve (AUC) with Image J.

We used the biotinylated oligos as bait for the PRRs IFI16 and cGAS, first pulling down the oligos using streptavidin beads and then assessing PRR–oligo interactions using western blotting. We first tested this assay using the published sequences of the ssDNA1, 2 and 3 oligos from the HIV-1 LTR as positive controls since they have already been shown to alter IFI16 activity (Jakobsen et al., 2013). The read-out for the *in vitro* biotin-streptavidin assay is the detection of IFI16 on a western blot. Since IFI16 is expressed as three isoforms, owing to an alternative splicing altering the spacer region between the two HIN domains (Johnstone et al., 1998), we typically detected three bands on the blots with apparent molecular masses between 89 and 98 kDa. While we did not distinguish between the three isoforms, the 95 kDa band was the most prominent and is likely to be the B form; this is consistent with the finding that the B isoform is the most abundantly expressed of the three (Jakobsen and Paludan, 2014). All three ssDNA oligos were able to bind to IFI16, with ssDNA2 showing the greatest intensity on the western blot (Figure 2A, top panel, lane 3). Interestingly, the oligo that showed the greatest ability to induce IFN, ssDNA1 (lane 2), did bind to IFI16 but not as strongly as ssDNA2, suggesting the intensity of binding does not correlate with this IFI16 function. Finally, the ssDNA3 oligo also bound to IFI16 but the band was much weaker (lane 4). As a negative control, a pulldown was performed with no oligo added and no IFI16 was detected (lane 5). The input lysate from this experiment showed a high level of IFI16 expression in the cells (lane 1). An additional lysate previously shown to contain IFI16 was used as a western blotting control (lane 6). The same blot was reprobed for cGAS, which was clearly detected in the lysate at approximately 54 kDa (Figure 2A, lower blot, lane 1) and the positive control lysate (lane 6). cGAS was not detected in any of the pulldowns with the ssDNA oligos (lanes 2, 3, and 4). The intensity of the bands was quantified using Image J (Figure 2, 3).

### IFI16 Binds to Wild-Type and Scrambled HK2 ssDNA

Next, we tested several HK2 oligos to determine if they could also bind to IFI16. We did pilot studies with potential oligos from within a HK2 *env* consensus sequence, one of which (HK2 *env*) was found to interact with IFI16 (Figure 2B, top panel, lane 4). Using mfold to model the secondary structure of the oligo, it predicted a stem-loop which we hypothesized is recognized by IFI16. To test this, we mutated ten bases (45–51) to remove the predicted stem-loop but found that the oligo was still able to bind to IFI16 (HK2 *env* mut, lane 5). In addition, we scrambled the sequence which altered the predicted secondary structure to a more open conformation and it bound to IFI16 with greater affinity than the original oligo (HK2 *env* scrambled, lane 6). It is possible that the predicted structures are not found in solution and thus the structure detected by IFI16 could be distinct from the mfold predictions. In addition, the preference of IFI16 for certain secondary structures, such as superhelical and cruciform (Brázda et al., 2012), might not preclude binding to others. We also identified oligos from the HERV-K113 LTR (lane 7) and *env* (lane 8), which had predicted secondary structures, with the former more stem-loop structures than the latter (Supplementary Figure S1, compare D to A). Both of these bound to IFI16 but HERV-K113 LTR did so with greater affinity than the *env* oligo. The ssDNA1 oligo was used as a positive control in these pulldown experiments (Figure 2B, lane 3). Further, IFI16 showed a high affinity for binding to a randomly generated oligo with high GC content and this oligo was included for comparison purposes (lane 2). Finally, IFI16 was detected in the control lysate (lane 9). Again, we reprobed the blot for cGAS and found that this was only detected in the experimental (Figure 2B, lower panel, lane 1) and control (lane 9) lysates; it was not detected in any of the pulldowns. We then used five-fold excess unlabeled oligo to compete with the biotinylated oligo for binding to IFI16. In this case, we chose to test two of the oligos that showed the strongest interaction with IFI16, ssDNA1 and HERV-K113 LTR. Addition of the unlabeled ssDNA1 oligo resulted in the loss of the band observed when the biotinylated oligo alone was used (Figure 2C, lane 2 versus lane 3); the same was found with the unlabeled HK113 LTR oligo (compare lane 4 versus lane 5).

Next, we took a more systematic approach to selecting oligos from the three major ORFs of the HERV-K provirus, as well as the LTR. We additionally considered sequence composition and predicted transcription factor sites as subjective criteria for ensuring diversity among the oligos that were tested. The regions were strategically chosen to represent a range of GC contents, coding potential, CpG islands and predicted transcription factor signals (Figure 1). Of these, the pulldowns with Peach (lane 5) showed the strongest interaction with IFI16, followed by Carrot (lane 8) and Cherry (lane 14) oligos (Figure 3, top panel). Both Peach and Carrot are from the LTR, while Cherry is from the *gag/pro/pol* ORF. In addition, the Kiwi oligo (lane 4) which was derived from the *env* sequence, showed a strong interaction with IFI16. Comparing the mfold structures, Peach and Carrot show similar elongated structures with multiple stem-loops, whereas both Cherry and Kiwi have more open conformations (Supplementary Figure S2). The HK2 *env* oligo overlaps partially with the Kiwi sequence although this was unintentional; the Kiwi oligo was from the HK113 locus alone, whereas the HK2 *env* oligo was derived from a consensus sequence of multiple loci belonging to the HK2 family (Hurst et al., 2016). None of these oligos were found to interact with cGAS (Figure 3, bottom panel, lanes 2–14).

### Innate Immune System Components in Teratocarcinoma Cells and hESCs

**FIGURE 4:**
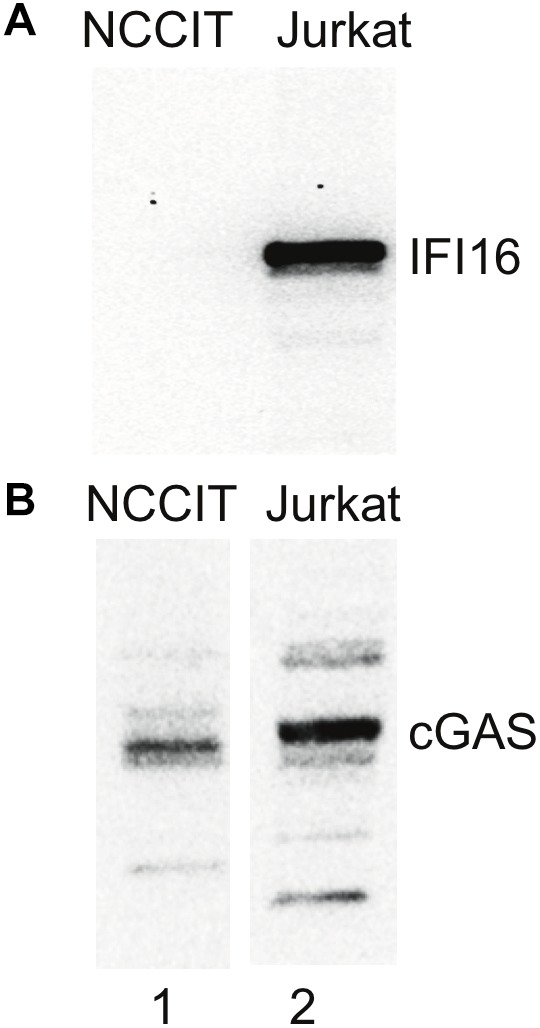
No IFI16 detected in NCCIT cells. NCCIT and Jurkat cells were lysed according to the method used in the pulldown experiments. The samples were then analyzed by SDS-PAGE and western blotting for IFI16 and cGAS. **(A)** IFI16 is not detected in NCCIT (lane 1) but is highly expressed in Jurkat cells (lane 2). **(B)** In contrast, cGAS is detected in both NCCIT (lane 1) and Jurkat (lane 2) cell lines. Aliquots of the same samples were used in panels **(A,B)** but loaded on separate SDS-PAGE gels.

Given our hypothesis that IFI16 could control HK2 replication, we next examined cells in which HERVs are known to be highly expressed. We checked for the expression of innate immune components in NCCIT, using publicly available RNAseq data derived from NCCIT (SRA accession number: SRX037079, runs: SRR089638/SRR094918) we found that MAP3K7, NFKB2 and TNFRSF1A were active in both datasets, while IL1R1, NFKB1, TNFRSF1B and TRAF6 were present with basal transcriptional activity. None of these genes was totally silenced in the tested samples. Crucially, there was no significant IFI16 expression in the NCCIT RNAseq dataset described above (SRX037079), as all IFI16 derived transcripts showed basal transcription activity (<10 tpm). Indeed, the NCCIT cells were found not to express the IFI16 protein when tested by western blotting (Figure 4). Furthermore, checking multiple single-cell RNAseq datasets (SRA project accession: PRJNA153427) derived from human early embryos and ESCs (4-cells, 8-cells, morula, blastocyst, hESC) we also found no significant IFI16 expression. Thus, a feature of stem cells, as well as teratocarcinoma cell lines with stem cell-like phenotype, could be the repression of IFI16 expression potentially to favor HERV expression during this developmental stage.

## Discussion

Here we provide preliminary data in support of the hypothesis that IFI16, but not cGAS, can detect ssDNA oligos derived from HERV sequences. Of the oligos tested, one from within the HK113 LTR (Peach) showed the most intense signal on the blot, suggesting a higher affinity for IFI16. Another LTR-derived oligo (Carrot) also showed a relatively intense interaction with IFI16 although the band intensity was similar to that from an oligo derived from the *gag* ORF (Cherry). Oligos from within the HERV *env* ORF could also interact with IFI16 although this was weaker than that observed with the LTR and *gag*-derived oligos. This suggests that sequences located within the HERV LTRs could be particularly strong ligands for IFI16, which may reflect a higher affinity for the type of structural elements within the HERV LTR. By analogy, the LTR of HIV-1 is highly structured; in the RNA genome, this structure is essential for reverse transcription (RT) (Harrich et al., 2000) and presumably similar folding can occur with the ssDNA produced during RT. To our knowledge, comprehensive analysis of the folding of the HERV LTR (either for the RNA transcript or as ssDNA produced during RT) has not been reported. Thus, it is impossible to speculate on the function, if any of HERV LTR secondary structure. However, our modeling of the secondary structures using mfold does indicate differences between the LTR oligos (Supplementary Figures S1D, S2A,B) and the other oligos, with the former possibly having a more complex, stem-loop rich fold compared to the open loop of the latter.

To explore the potential of HERV cDNA to interact with IFI16, we deliberately used an *in vitro* system with biotinylated oligos which could subsequently be bound by streptavidin beads in our pulldowns. This meant only our biotinylated oligos were pulled down in the experiment and so we can be sure that where we detect IFI16 on the blot, it is because it bound to the biotinylated oligo and not gDNA. We also used unlabeled oligos to compete for binding to IFI16 with the biotinylated oligos, showing that we were specifically detecting this interaction in our pulldowns. While our system did not test HERV oligos produced *in vivo*, it nonetheless provides a proof of principle that HERV cDNAs can be ligands for PRRs. Importantly, we did not find an ssDNA oligo derived from HK2 that was completely negative in pulling down IFI16. Although there are plenty of possibilities that we have not tested, this suggests that IFI16 has a wide flexibility in binding ssDNA. It would be interesting in the future to identify ssDNA sequences that are unable to bind to IFI16 as these could possibly be escaping from the receptor.

While the foregoing does indicate the potential for HERV-derived ssDNA to act as a ligand for IFI16, it is not certain that HERV RNA transcripts are reverse transcribed in cells in modern humans. This is theoretically possible but it has not been directly reported and, indeed, is difficult to test given the risk of genomic DNA confounding the detection of cDNA. It is clear that HK2 has been proliferating in humans since the human-chimpanzee split (Marchi et al., 2014; Wildschutte et al., 2016), with HK2 retroviral integrations being polymorphic in modern humans as a result of full retroviral life cycles within the last million years. This supports the hypothesis that cDNA is produced as part of the retroviral life cycle; if so, then the interaction with IFI16 could have been important for controlling proliferation over this timescale of co-evolution. Thus, the interaction of IFI16 and HERV cDNA has potentially been important throughout recent human evolution. If HK2 died out during the last 250,000 years then these mechanisms would have been rendered redundant very recently with respect to controlling HERV activity.

We also found that another DNA receptor, cGAS, did not interact with any of the oligos tested. Importantly, cGAS is known to have a primary role in the detection of viral DNA leading to an IFN response (Sun et al., 2013). The sensing of DNA by IFI16 could fit in with the cGAS-cGAMP-STING axis possibly by acting as an amplifier of the signal (Hansen et al., 2014; Jønsson et al., 2017). The latter would be important for the production of IFN in response to HERV cDNA. However, IFI16 has other functions, namely pyroptosis and inflammasome activation, which could be activated following detection of HERV cDNA. This suggests that there are DNA species which may be ligands for one PRR and not the other, allowing diverse responses to different ligands. We suggest that the role of detecting retroviral DNA products represents an important function of IFI16. It could be that the detection of ssDNA by IFI16 leads to a non-inflammatory response (e.g., apoptosis) with the aim being to clear ERV elements without triggering inflammation. Thus, IFI16 might not be functionally redundant but could have a distinct role in detecting ssDNA such as that produced during endogenous retroviral reverse transcription. We propose that this suggests an evolutionary pressure on IFI16 for a more sensitive detection of retroviral-specific DNA (i.e., ssDNA), possibly leading to distinct outcomes compared to those resulting from cGAS-mediated signaling.

Using western blotting and RNA-Seq analysis, we found that an ESC line derived from a teratocarcinoma (NCCIT) did not express IFI16. Further, RNA-Seq analysis of ESCs also showed only basal (<10 tpm) levels of IFI16. The absence of IFI16 transcripts (NCCIT and ESCs) and protein (NCCIT) suggests that IFI16 is not expressed at this developmental stage. There could be reasons for this in terms of the regulation of the cell cycle by IFI16; it was found to slow progression through the cycle in hematopoietic stem cells (Piccaluga et al., 2015) and this could possibly be detrimental at the early stage of development. The absence of IFI16 could also be important in ESCs if it facilitates HERV expression which contributes to stem cell identity (Magiorkinis et al., 2017). Thus, the suppression of proteins including IFI16 could further promote HERV expression at this critical stage.

IFI16 has an additional role as a tumor suppressor (Ouchi and Ouchi, 2008) and thus its absence in NCCIT also removes an important check on tumorigenesis. This could have contributed to the transformation into teratocarcinoma cells. The tumor suppressor function is mediated via IFI16 associating with other proteins, particularly via the pyrin domain of IFI16 which facilitates protein–protein interactions (Supplementary Figure S3). Importantly, IFI16 can interact with the tumor suppressor protein p53 and can induce apoptosis in an NF-κB-dependent manner (Gugliesi et al., 2010). Since IFI16 is absent, the interaction with p53 would not be possible. Further, NCCIT cells have been reported to have defective p53 (Burger et al., 1998); a defect in this protein alone would contribute to tumorigenicity but the lack of IFI16 could be a second “hit” that ultimately leads to loss of cell cycle control and tumor formation. Where IFI16 is present, it could detect HERV cDNA and then interact with NF-κB to drive apoptosis. This would have the dual effect of dealing with aberrant HERV expression and killing off a cell before it can transform. In contrast, in the absence of IFI16, HERV expression goes unchecked and can be further enhanced such as by the binding of activated NF-κB to HERV LTRs. RNA-Seq analysis showed that this transcription factor is expressed in NCCIT. In contrast, another study in a different teratocarcinoma, Tera-1, found that a HERV LTR co-opted to regulate the PRODH gene exhibited an enhancer effect in responsive to Sox2 and not NF-κB (Suntsova et al., 2013), suggesting that NF-κB activation alone might not greatly alter HERV expression.

The teratocarcinoma cell line, NCCIT, is particularly permissive to HERV expression and this could be due to alterations in the epigenetic profile and the expression of stem cell factors. Teratocarcinoma cells are derived from germ cell tumors and were documented early in the study of HERVs to express retroviral particles (Löwer et al., 1984). By contrast, other cancer cell lines derived from somatic cells we have studied do not overexpress HERVs or at least not to the same high copy number we observed in the NCCIT. For example, Jurkat, which are derived from a T cell leukemia, did not show detectable HK2 in qPCR (data not shown) compared to NCCIT. Notably, Jurkat constitutively express IFI16 while NCCIT do not (Figure 4A), which could add support for a role for IFI16 in suppressing HK2 expression in somatic cells. In a microarray study, reproductive tissues were among the subset of body tissues found to express most of the active HERVs (Seifarth et al., 2005) and it is now well-known that HERVs have roles in the normal physiology of human reproduction (Soygur and Sati, 2016), including in stem cell identity (Magiorkinis et al., 2017). The expression of HERVs in early development is gaining recognition as a critical feature of normal ontogeny although it could also set the stage for adult diseases (Magiorkinis et al., 2017). Thus, it could be that heightened HERV expression in these cells reflects the normally increased level of transcription in this type of tissue.

In order to further examine this hypothesis, we propose examining the effects of overexpression of IFI16 in the NCCIT cells. Among other read-outs, we suggest measuring HERV transcription using RT-qPCR assays with samples from wild-type and IFI16-expressing NCCITs. In addition, the downstream consequences of IFI16 activity could be explored. For example, expression and cleavage of IL-1β could be detected using western blotting in order to assess IFI16-induced inflammasome activation. Cell viability could be assessed to determine if NCCIT expressing IFI16 are more susceptible to cell death, likely via IFI16-mediate pyroptosis. Alternatively, we could use shRNA to knockdown IFI16 in Jurkat cells and then measure HERV expression.

One difficulty with the preliminary data included herein is the absence of evidence using physiological HERV cDNA. We have explained the technical difficulties in doing so above. However, further support for this hypothesis would come from showing that (1) HERV cDNA is produced and (2) that IFI16 could bind to this cDNA, resulting in activation of IFI16-mediated signaling. Overcoming the difficulty of isolating ssDNA uncontaminated by gDNA and establishing that this has been reverse transcribed from HERV loci would be a major step in understanding the activity of HERVs.

## Conclusion

While it is known that HERVs are transcribed, it is unclear whether HERV cDNA is made in present-day humans and under which circumstances. If HERV cDNA is produced, it could be immunogenic and we have shown that it could be a ligand for IFI16. Given the presence of multiple HERV loci in the genome, it is difficult to study the interaction of HERVs with components of the innate immune response in a targeted way, especially when the molecule of interest is DNA. In this hypothesis paper, we have used an *in vitro* assay to explore if IFI16 can bind to ssDNA, and more specifically HERV oligos, particularly those derived from the HK2 LTR. This would suggest that, at least in the past when HERV ssDNA was produced in cells, it was an endogenous ligand for IFI16. Moreover, we have found that NCCIT, which exhibit high HK2 expression, do not express IFI16. Interestingly, IFI16 was also absent in ESCs and it is tempting to speculate that the lack of IFI16 contributes to stem cell identity. This could be by allowing unchecked HERV expression in order to facilitate the expression of those that function in stem cell identity (Santoni et al., 2012; Chiappinelli et al., 2015). In contrast, HERVs are normally suppressed in somatic cells and, if our hypothesis is verified, IFI16 could act as a check on proliferation in these cells. Putative roles for HERVs in various cancers have been described, including a recent paper describing the oncogenic properties of HK2 *env* (Lemaître et al., 2017). Thus, the absence of IFI16 and unchecked HK2 expression could permit cellular transformation through the action of HERV proteins. In contrast, IFI16 is a potentially important sentinel for aberrant HERV expression in somatic cells, preventing the onset of oncogenesis.

## Author Contributions

GM and TH designed and performed the research and wrote the first draft of the manuscript. TK contributed to the analysis of RNA-Seq data and AA designed the oligos in Figure 3. AK and AS contributed materials, designed and discussed experimental data. All authors commented on drafts and approved the final manuscript.

## Conflict of Interest Statement

The authors declare that the research was conducted in the absence of any commercial or financial relationships that could be construed as a potential conflict of interest.

## Supplementary Material

FIGURE S1 Predicted secondary structures of oligos used in the pilot study. Oligos used in Figure 2 were analyzed using mfold by inputting the oligo sequences into the online server, using the default parameters for DNA. This typically generated three or more possible structures based on the free energy (ΔG) (Zuker, 2003). We have selected one image for each of four oligos: **(A)** HK2 *env*, **(B)** HK2 env mut, **(C)** HK2 env Scrambled 2 and **(D)** HK113 LTR.

FIGURE S2 Predicted secondary structures of the HK113 oligos. The oligos showing the strongest interaction with IFI16 on the western blot were analyzed using mfold. The structure predictions were obtained by inputting the oligo sequences into the online mfold server, using the default parameters for DNA. This typically generated three or more possible structures based on the free energy (ΔG) (Zuker, 2003). We have selected one image for each of four oligos: **(A)** Peach, **(B)** Carrot, **(C)** Cherry and **(D)** Kiwi.

FIGURE S3 Model of IFI16 protein–protein interactions. Known and predicted interactions of IFI16 with other cellular proteins, including the IFN signaling pathway members TBK1 and IRF3. Importantly, IFI16 interacts with key cell cycle and tumor suppressor proteins such as p53, indicating that its functions extend beyond the innate immune signaling pathway. The STRING database (Szklarczyk et al., 2017) was used to model the interactions with human IFI16.
