# Supplementary figures and images for: Interferon-Inducible Protein 16 (IFI16) Has a Broad-Spectrum Binding Ability Against ssDNA Targets: An Evolutionary Hypothesis for Antiretroviral Checkpoint
Source: Front Microbiol. 2019 Jul 4;10:1426. doi: 10.3389/fmicb.2019.01426 (PMC6621918; doi:10.3389/fmicb.2019.01426)

A

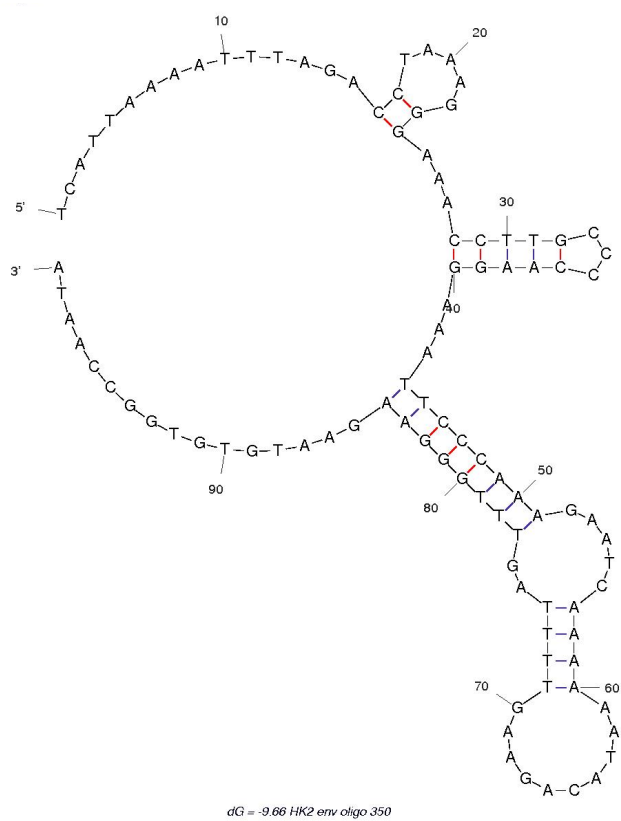

B

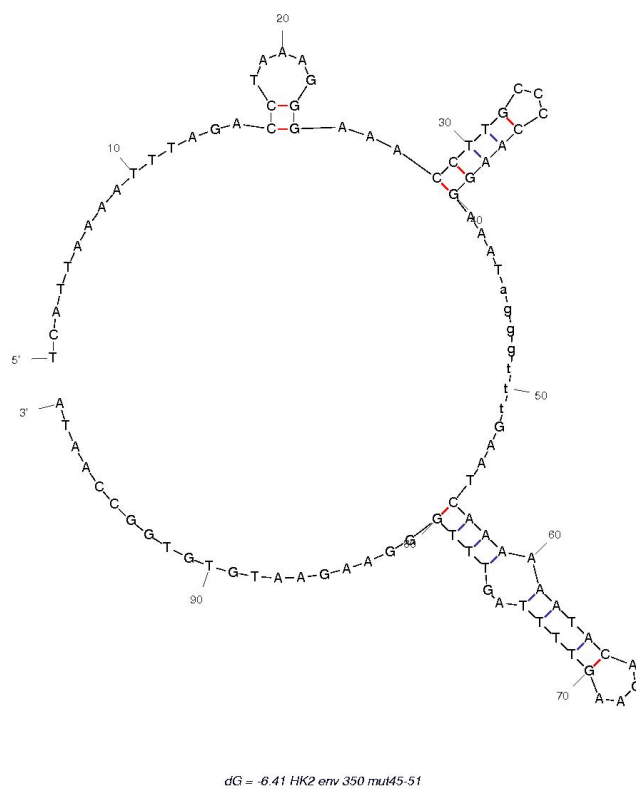

C

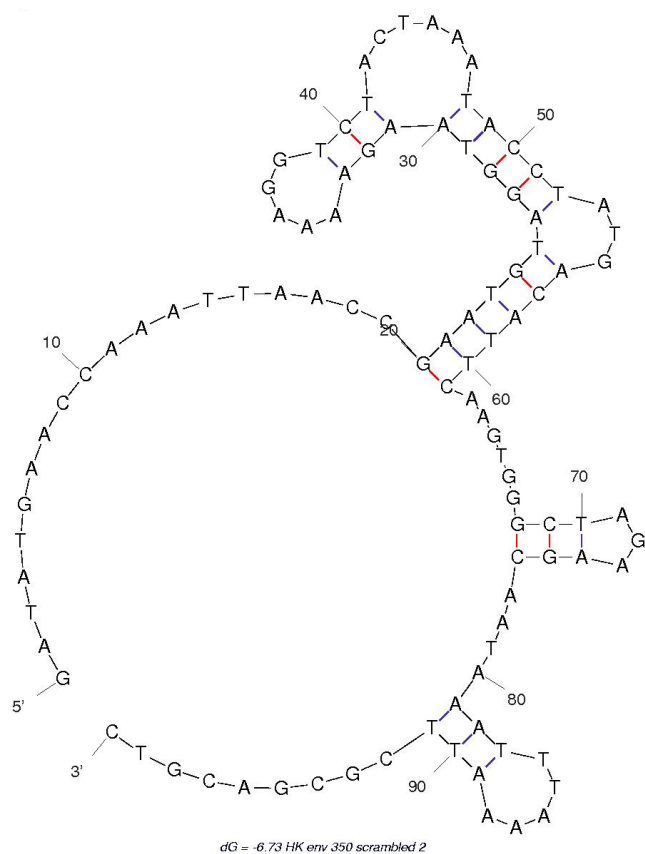

D

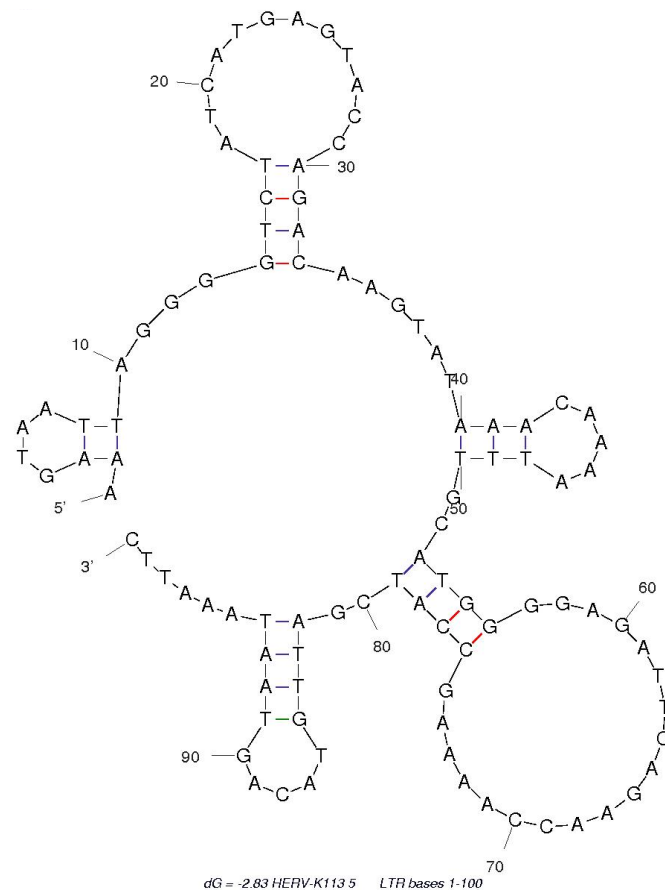

Supplement: FIGURE S1 — Predicted secondary structures of oligos used in the pilot study. Oligos used in Figure 2 were analyzed using mfold by inputting the oligo sequences into the online server, using the default parameters for DNA. This typically generated three or more possible structures based on the free energy (ΔG) (Zuker, 2003). We have selected one image for each of four oligos: (A) HK2 env, (B) HK2 env mut, (C) HK2 env Scrambled 2 and (D) HK113 LTR. [file Data_Sheet_1.PDF]

A

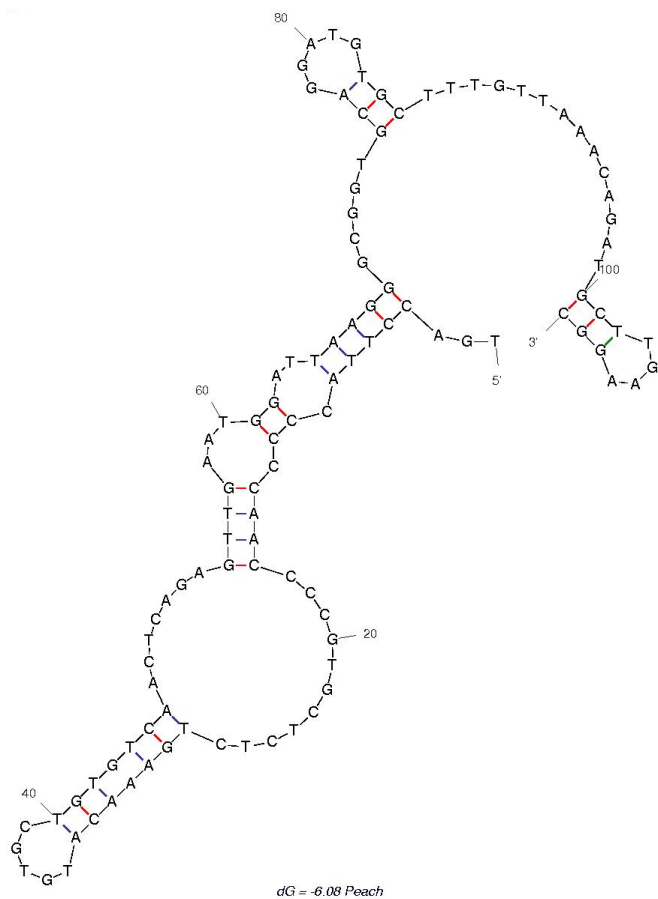

B

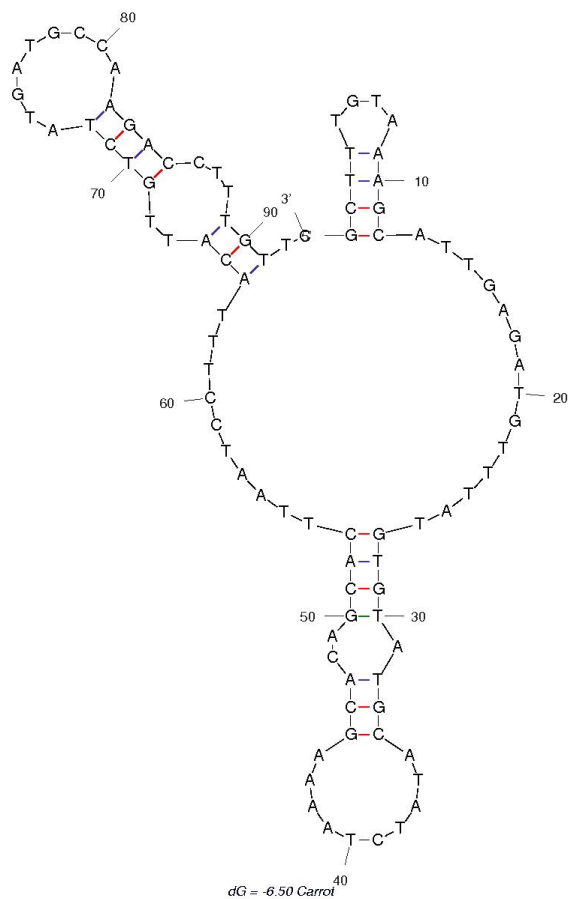

C

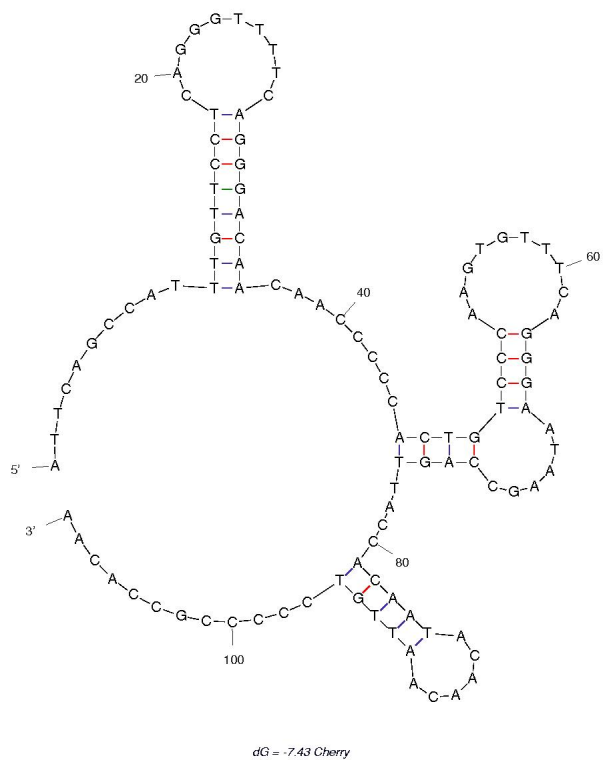

D

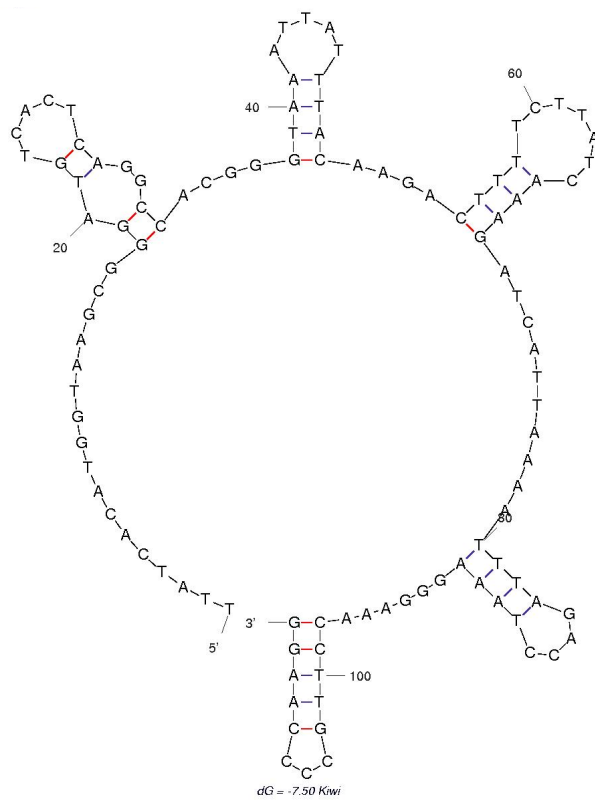

Supplement: FIGURE S2 — Predicted secondary structures of the HK113 oligos. The oligos showing the strongest interaction with IFI16 on the western blot were analyzed using mfold. The structure predictions were obtained by inputting the oligo sequences into the online mfold server, using the default parameters for DNA. This typically generated three or more possible structures based on the free energy (ΔG) (Zuker, 2003). We have selected one image for each of four oligos: (A) Peach, (B) Carrot, (C) Cherry and (D) Kiwi. [file Data_Sheet_2.PDF]

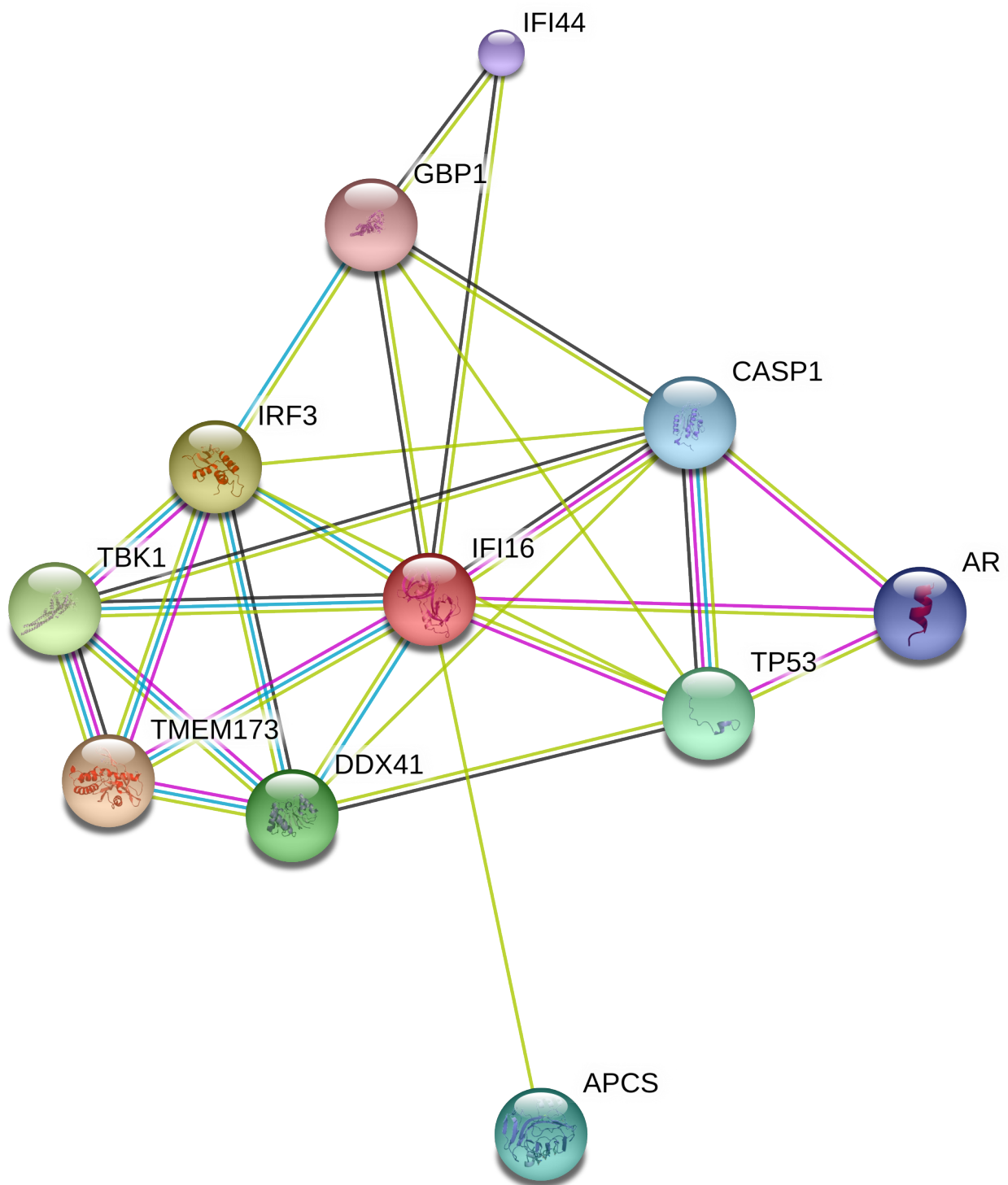

Supplement: FIGURE S3 — Model of IFI16 protein–protein interactions. Known and predicted interactions of IFI16 with other cellular proteins, including the IFN signaling pathway members TBK1 and IRF3. Importantly, IFI16 interacts with key cell cycle and tumor suppressor proteins such as p53, indicating that its functions extend beyond the innate immune signaling pathway. The STRING database (Szklarczyk et al., 2017) was used to model the interactions with human IFI16. [file Data_Sheet_3.PDF]
